# Cyclic adenosine monophosphate/phosphodiesterase 4 pathway associated with immune infiltration and PD-L1 expression in lung adenocarcinoma cells

**DOI:** 10.3389/fonc.2022.904969

**Published:** 2022-08-01

**Authors:** Ling Tong, Minjie Shan, Wen Zou, XianLing Liu, Dean W. Felsher, Jingjing Wang

**Affiliations:** ^1^ Division of Oncology, Departments of Medicine and Pathology, Stanford University School of Medicine, Stanford, CA, United States; ^2^ Department of Oncology, The Second Xiangya Hospital of Central South University, Changsha, China

**Keywords:** programmed cell death-ligand 1, cyclic adenosine monophosphate, immune infiltration, MYC, lung adenocarcinoma, the cancer genome atlas

## Abstract

**Background:**

The cyclic adenosine monophosphate/phosphodiesterase 4 **(**cAMP/PDE4) pathway is involved in inflammation and immune regulation; however, the effect of cAMP/PDE4 on immune infiltration and immune evasion in lung adenocarcinoma (LUAD) remains unclear.

**Methods:**

CBioPortal, which is the The Cancer Genome Atlas (TCGA) online database, and the Kaplan Meier plotter were used to analyze the association between genes and the prognosis of TCGA-LUAD. Tumor Immune Estimation Resource (TIMER) was used to analyze the association between gene expression and immune infiltration. The Genecards database was used to identify the transcription factors of related genes. The lung adenocarcinoma cell line H1299 and A549 were treated with cAMP pathway drugs. Flow cytometry and qRT-PCR were used to detect the PD-L1 protein and gene expression, respectively. A one-way analysis of variance with Tukey’s *post-hoc* test or a Student’s t-test were used.

**Results:**

It was found that PDE4B and CREB1, which are downstream genes of the cAMP/PDE4 axis, were differentially expressed in LUAD and adjacent tissues and are correlated with the prognosis and immune infiltration of LUAD. In the CBioPortal database, cAMP pathway genes are closely related to programmed cell death-ligand 1 (PD-L1) expression in TCGA-LUAD. The protein-protein interaction revealed that there was a direct interaction between CREB1/CREBBP, which are the downstream molecules of the cAMP/PDE4 axis, and MYC; additionally, MYC was predicted to bind to the PD-L1 transcription site and regulate PD-L1 expression. CREB1 was also predicted to transcriptionally bind to both MYC and PD-L1. These results predicted the interaction network of cAMP/PDE4/CREB1/CREBP/MYC/PD-L1, and the core factor may be related to MYC. In the cell experiment, forskolin (an adenylate cyclase activator) and zardaverine (a PDE4 inhibitor) enhance the cAMP pathway and decrease PD-L1 expression, while SQ2253 (an adenylate cyclase inhibitor) inhibits the cAMP pathway and increases PD-L1 expression of the LUAD cell lines H1299 and A549, and MYC regulation by these drugs was positively correlated with PD-L1 regulation, which verified the regulation of the cAMP/PDE4 pathway on MYC and PD-L1.

**Conclusions:**

This study showed that the cAMP/PDE4 pathway may play an important role in PD-L1 regulation and immune infiltration in LUAD.

## Introduction

Programmed cell death-ligand 1 (PD-L1), also known as B7 homolog 1 (B7-H1) or cluster of differentiation 274 (CD274), is a co-inhibitory ligand of programmed cell death protein-1 (PD-1) (1). Inhibition of PD-1/PD-L1 could play a role in the treatment of various cancers, including lung cancer. However, the sensitivity of patients to PD-1/PD-L1 inhibitors is highly variable. Interestingly, patients with chronic obstructive pulmonary disease (COPD) are more sensitive to PD-1/PD-L1 inhibitors (2), which may be related to the changes in immune micro-environment secondary to the disease process. The role of the cyclic adenosine monophosphate/phosphodiesterase 4 **(**cAMP/PDE4) axis in immune system regulation is supported by the Food and Drug Association as a treatment for COPD (3). However, the role of the cAMP/PDE4 axis in antitumor immunity remains unclear. The expression of PD-L1 is an important component of antitumor immunity and an important biomarker for predicting PD-1/PD-L1 inhibitors (4). Therefore, it is of great significance to explore the effects of the cAMP/PDE4 axis on PD-L1 expression and the immune micro-environment in patients with lung cancer.

cAMP is the first intracellular second messenger that mediates the physiological regulation of extracellular signals. It primarily acts on downstream effector molecules, such as cAMP-dependent protein kinase A (PKA) and cyclic adenosine monophosphate effector binding protein (CREB), to regulate cell proliferation, secretion, metabolism, and apoptosis. PDE4 can hydrolyze cAMP, regulate its intracellular concentration, and affect the physiological function of the cAMP axis (5). Increasing cAMP in tumor cells by inhibiting PDE4 activity can promote the apoptosis of both solid and blood tumor cells, as well as inhibit invasion, migration, and angiogenesis (6–8). The cAMP/PDE4 axis can also inhibit innate and adaptive immune cells activity, as well as the production of pro-inflammatory cytokines (9–12). The cAMP/PDE4 pathway is thus involved in the pathogenesis of various inflammatory diseases, including COPD, asthma, psoriasis, and rheumatoid arthritis. The cAMP/PDE4 axis is an important mediator of inflammation and immune regulation and a potentially important therapeutic target for these conditions.

In the field of antitumor immunity, it was found that a decrease in cAMP concentration in the lactic acid environment of lung cancer cells inhibits PKA activity, leading to the activation of transcription activator PDZ-binding motif (TAZ) and the promotion of PD-L1 expression through the interaction between TAZ and transcription factor terminating extended area descriptor (TEAD) (13). In diffuse large B-cell lymphoma (DLBCL), cAMP regulates the transduction mechanism of JAK/STAT and increases PD-L1 expression through the autocrine loop produced by cytokines (14). The differences among tumors in how cAMP regulates PD-L1 expression may be related to different regulatory mechanisms.

This study explored the effect of cAMP/PDE axis regulation on PD-L1 expression and immune infiltration in patients with lung cancer to evaluate the role of the cAMP/PDE4 axis on tumor immune evasion.

## Materials and methods

### Materials

The adenylate cyclase activator forskolin [HY-15371], PDE4 inhibitor zardaverine [HY-15485], adenylate cyclase inhibitor SQ22536 [HY-100396] and BET inhibitor JQ1 [HY-78695] were purchased from MedChemExpress. Human IFN-gamma [285-IF) were purchased from R&D systems (Minnesota, USA); Forskolin, zardaverine, and JQ1 were dissolved in dimethyl sulfoxide (DMSO) and stored at -20°C.

### Cell lines and cell culture

The lung adenocarcinoma (LUAD) cell lines H1299 and A549 were purchased from the American Type Culture Collection (Manassas, VA, USA) and cultured at 37°C in 5% CO_2_ in a Roswell Park Memorial Institute-1640 medium (Thermo Fisher Scientific, Waltham, MA, USA) and supplemented with 10% fetal bovine serum and 1% penicillin-streptomycin.

### Flow cytometric analysis

Twenty-four hours after treatment initiation, the media were removed, and the cells were washed with ice-cold phosphate-buffered saline (PBS). The cells were trypsinized, collected, and washed with PBS to remove residual trypsin and subsequently incubated with PE Anti-PD-L1 antibody (ab270652, Abcam) or isotype control antibodies (Abcam, Cambridge, UK) for 30 min at 4°C. Next, they were washed with PBS before being fixed in 1 % paraformaldehyde. PD-L1 surface expression was measured using a BD Accuri C6 flow cytometer (BD Biosciences, San Jose, CA, USA). Data were analyzed with FlowJo v10.6.2 (FlowJo LLC, Ashlang, OR USA), and all experiments were conducted in triplicates.

### Quantitative real-time polymerase chain reaction

RNA was isolated from the cell lines using a TRIzol reagent (Invitrogen, Waltham, MA). cDNA was synthesized using SuperScript III (ThermoFisher). qRT-PCR was performed using the SYBR Green qRT-PCR Kit (Roche, Basel, Switzerland) and an Applied Biosystems Real-Time PCR System (Life Technologies, Carlsbad, CA) with QuantStudio 12 K Flex Software to detect PD-L1, MYC, CD47, and ubiquitin-conjugating enzyme (UBC) mRNA expressions. UBC was used as a reference gene. Relative gene expression was calculated using the 2^−ΔΔCt^ method. All experiments were conducted in triplicates. The primer sequences used are listed in **Supplementary Table 1**.

### Prognostic analysis based on the public database

The Kaplan–Meier plotter (http://kmplot.com/) software was used to analyze the prognosis of patients with high- and low-gene expression in The Cancer Genome Atlas Lung Adenocarcinoma (TCGA-LUAD), Gene Expression Omnibus (GEO), and European Genome-phenome Archive (EGA) databases.

### Gene correlation analysis

CBioPortal (http://www.cbioportal.org/), which is the online database of TCGA, was used to analyze the associations between genes in TCGA-LUAD. The Kyoto Encyclopedia of Genes and Genomes (KEGG) (http://www.kegg.jp/) was used to display genes related to the cAMP pathway. A Venn diagram (http://bioinformatics.psb.ugent.be/webtools/Venn/) was used to determine the intersection of the different gene groups. The Genecards database (https://www.genecards.org) was used to identify the transcription factors of related genes.

### Prognostic analysis based on the public database

Gene expression comparisons between LUAD tumors and normal controls were investigated through UALCAN (http://ualcan.path.uab.edu/) with two independent sample T tests. The Kaplan–Meier plotter (http://kmplot.com/) software was used to analyze the prognosis of patients with high- and low-gene expression in the TCGA-LUAD, GEO, and EGA databases.

### Immune infiltration analysis

Tumor Immune Estimation Resource (TIMER; cistrome.shinyapps.io/timer) was used to analyze the association between gene expression and immune cell infiltration in LUAD samples, including B cells, CD4+ T-cells, CD8+ T-cells, neutrophils, macrophages, and dendritic cells.

### Protein-protein interaction network

The PPI network was constructed using the STRING database [http://string-db.org) (15)]. The confidence score was set at >0.4.

### Statistical analysis

Statistical analysis was performed using GraphPad Prism V8.0 software (GraphPad Software Inc., San Diego, CA). Continuous numerical data were described as the mean ± standard error. A one-way analysis of variance with Tukey’s *post-hoc* test or a Student’s t-test (two-tailed, equal variance) were used to compare the effects of different drug treatments targeting the cAMP pathway in LUAD cell lines. Statistical significance was set at P<0.05.

## Results

### Expression of cAMP/PDE4 axis gene and its relationship with prognosis in LUAD

**Figure 1:**
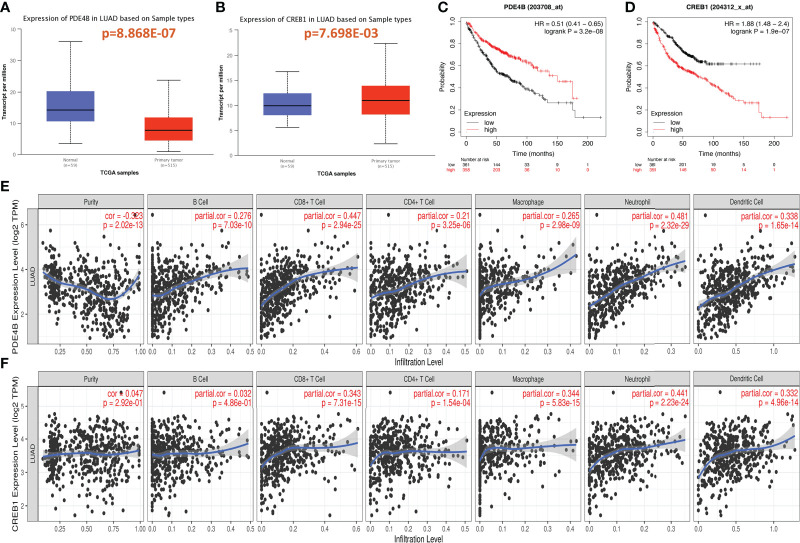
Expression of cAMP/PDE4 pathway genes and its effect on prognosis and immune infiltration in lung adenocarcinoma. **(A, B)**: Differential expression of PDE4B and CREB1 in lung adenocarcinoma and normal control tissues. **(C, D)**: PDE4B and CREB1 were analyzed for prognosis using the Kaplan–Meier plotter database. **(E, F)**: PDE4B and CREB1 were analyzed for immune infiltration using the TIMER database in TCGA-LUAD.

PDE4B and CREB1, which are downstream genes of the cAMP/PDE4 axis, were analyzed in terms of differential expression in LUAD and adjacent tissues in TCGA-LUAD, and the differential expression was evaluated by a T test. Compared to the Para cancerous control, PDE4B was lower expressed, and CREB1 was higher expressed in LUAD (**Figures 1A, B**).

The Kaplan–Meier plotter database was then used to analysis the relationship between PDE4B and CREB1 and prognosis in LUAD. PDE4B (p=3.2e-08) and CREB1 (p=1.9e-07) were significantly correlated with LUAD prognosis. High PDE4B expression and low CREB1 expression were associated with better patient survival and prognosis (**Figures 1C, D**).

### Relationship between cAMP/PDE4 axis genes and immune infiltration in LUAD

The relationships between the cAMP/PDE4 pathway genes and immune infiltration were explored using the TIMER database. PDE4B was significantly associated with the infiltration of B cells (p=7.03e-10), CD8+ T-cells (p=2.94e-25), CD4+ T-cells (p=3.25e-06), macrophages (p=2.98e-09), neutrophils (p=2.32e-29), and dendritic cells (p=1.65e-14) (**Figure 1E**). CREB1 was significantly associated with the infiltration of CD8+ T-cells (p=7.31e-04), CD4+ T-cells (1.54e-04), macrophages (p=5.83e-15), neutrophils (p=2.23e-24), and dendritic cells (p=4.96e-14) (**Figure 1F**). Both the PDE4B and CREB1 genes are related to the markers of immune cells in LUAD. Particularly, PDE4B is closely related to the markers of most immune cells (**Table 1**). These results suggest that the cAMP/PDE4 pathway may regulate immune cell infiltration in LUAD.

**Table 1:** Correlation analysis between *CREB1* and *PDE4B* and relate markers of immune cells.

Cell type	Gene marker	CREB1		PD4B	
		Pearson	P	Pearson	P
B cell	CD19	-0.03	0.543	0.36	8.65e-17
	MS4A1	0.08	0.071	0.32	5.23e-14
CD8+ T Cell	CD8A	0.06	0.143	0.34	4.23e-15
	CD8B	0.04	0.395	0.26	5.07e-9
Th1	CXCR5	0.02	0.611	0.32	2.83e-13
	ICOS	0.13	0.0028	0.35	2.9e-16
	STAT1	0.19	1.5e-5	0.19	2.07e-5
Th2	CCR3	-0.12	5.9e-03	0.13	3.9e-3
	STAT6	-0.08	0.072	-0.06	0.161
Th17	IL-23R	0.17	7.5e-05	0.21	2.5e-6
	STAT3	0.18	3.6e-05	0.04	0.315
Treg	FOXP3	0.02	0.599	0.26	1.78e-9
	CCR8	0.19	1.04e-05	0.34	1.55e-15
	STAT5B	0.24	5.91e-8	0.26	4.56e-9
Macrophage	CD68	0.04	0.328	0.016	0.28
	CD84	0.33	4.5e-14	0.239	1.46e-10
	MS4A4A	0.16	2.69e-04	0.34	1.93e-15
	CD163	0.19	1.66e-05	0.33	7.97e-15
Monocyte	CD14	-0.01	0.77	0.29	3.8e-11
	C3AR1	0.2	4.39e-6	0.34	2.64e-15
	CSF1R	0.18	5.7e-5	0.31	9.95e-13
NK	XCL1	0.05	0.218	0.16	3.92e-4
	NCR1	0.08	0.059	0.27	1.15e-9
	FPR1	0.16	2.5e-4	0.42	1.39e-23
	SIGLEC5	0.18	2.83e-5	0.27	3.06e-10
	FCGR3B	0.18	3.65e-5	0.27	9.29e-10
DC	CCL13	0.15	0.001	0.22	5.04e-7
	CD209	0.22	3.77e-7	0.3	2.32e-12

### cAMP pathway genes are closely related to PD-L1

**Figure 2:**
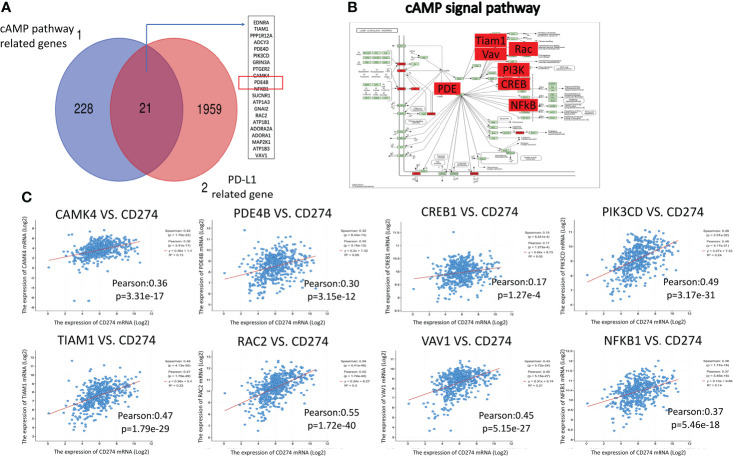
Correlation analysis between cAMP pathway genes and PD-L1. **(A)**: A Venn diagram was mapped for the cAMP pathway and PD-L1-related gene groups. There were 21 overlapping genes. **(B)**: The overlapping genes were placed in the KEGG cAMP pathway diagram (red-labeled genes). **(C)**: TCGA online visual database CBioPortal was used to analyze the correlation between cAMP pathway genes and PD-L1, which showed a close relationship.

In total, 249 genes related to the cAMP pathway were found on the KEGG pathway website. In addition, 1,980 genes related to PD-L1 expression in LUAD were found on CBioPortal (assuming Spearman’s correlation ≥0.3, P<0.05). These two gene groups were mapped using a Venn diagram, and 21 genes considered to be related to the cAMP and PD-L1 pathways overlapped (**Figure 2A**). These overlapping genes were placed in the KEGG cAMP pathway diagram, and their positions in the cAMP pathway were determined (**Figure 2B**, red-labeled genes), with the PDE4B gene at the core position. Data from CBioPortal was used to analyze the associations between the cAMP pathway core gene mRNA expression and PD-L1. The expression of *PDE4B, CREB1, CAMK4, PIK3CD, TIAN1, RAC2, VAV1*, and *NFKB1*were all found to be closely related to PD-L1 expression in LUAD (Pearson correlation coefficient > 0.15, P<0.05) (**Figure 2C**).

### Downstream molecules of the cAMP pathway regulate MYC, which can regulate PD-L1 transcription

**Figure 3:**
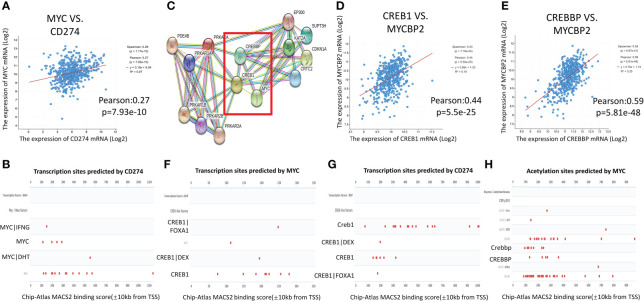
Interaction network of cAMP/CREB1/CREBP/MYC/PD-L1. **(A)**: MYC was positively correlated with PD-L1 gene expression in TCGA-LUAD (CBioPortal). **(B)**: The transcription factors of PD-L1 were queried in the Genecards database. MYC had transcriptional binding with PD-L1 in Chip-seq report. **(C)**: The PPI analysis between downstream molecules of the cAMP pathway and MYC was performed using the STRING website, and their co expression scores. **(D, E)**: CREB1 and CREBBP were positively correlated with MYC binding protein 2 expression in TCGA-LUAD (CBioPortal). **(F, G)**: Genecards gene database was used to predict the transcription factors of MYC and PD-L1. Chip-seq report predicted that CREB1 had transcriptional binding with MYC and PD-L1. **(H)**: The Chip-seq report predicted that CREBBP bound to the acetylation site of MYC.

A previous study confirmed that MYC can directly bind to the promoter of PD-L1 of tumor cells, regulate its transcriptional activity, and participate in tumor immune evasion (16). Based on data from CBioPortal, mRNA expression of PD-L1 was associated with MYC expression in LUAD (r=0.27, p=7.93e-10) (**Figure 3A**). The Genecards database was used to predict the activity of the PD-L1 transcription factors. MYC had a transcriptional binding to PD-L1 (**Figure 3B**).

The PPI analysis on the STRING website showed that there was a direct interaction between CREB1/CREBBP, which is the downstream molecule of the cAMP/PDE4 axis, and MYC (**Figure 3C**). The correlation score between MYC and CREBBP is 0.991. The correlation score between CREB1 and MYC is 0.653. The mRNA expression of CREB1 (r=0.44, p=5.5e-25) and CREBBP (r=0.59, p=5.81e-48) were strongly associated with MYC binding protein 2 (MYCBP2) expression in TCGA-LUAD database (**Figures 3D, E**). In the Genecards database, CREB1 showed transcriptional binding to both MYC and PD-L1 (**Figures 3F, G**). The Chip-seq report also found that CREBBP bound to the acetylation site of MYC (**Figure 3H**). These results predicted the interaction network of cAMP/PDE4/CREB1/CREBP/MYC/PD-L1, and key regulatory factor may be related to MYC.

### The correlation between the cAMP/PDE4 axis and PD-L1 was verified by a cell experiment

**Figure 4:**
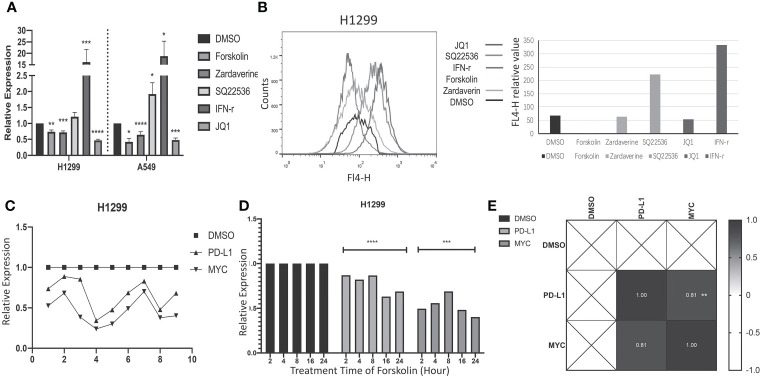
The cAMP pathway regulates the expression of PD-L1 and MYC in a cell experiment. **(A)**: qRT-PCR was used to detect the expression of the PD-L1 gene in the H1299 and A549 lung adenocarcinoma cell lines after treatment with forskolin, zardaverine, JQ1, SQ2253, and IFN-gamma. The drug treatment groups showed significant differences when compared to the control group. **(B)**: PD-L1 protein expression in H1299 cells treated with forskolin, zardaverine, JQ1, SQ2253, and IFN-γ was detected using flow cytometry. There was a significant difference between the groups (P < 0.001). **(C)**: The mRNA expression of PD-L1 and MYC in multiple H1299 cell samples treated with forskolin was detected by qRT-PCR. **(D)**: The gene expression of PD-L1 and MYC in the H1299 cell line treated with forskolin for 2, 4, 8, 16, and 24 hours was detected by qRT-PCR. The comparison between different time points revealed significant differences (p<0.001). **(E)**: Correlation analysis of PD-L1 and MYC mRNA expression in H1299 cells treated with forskolin. In **(A, D)**: data shown are mean ± SD of the triplicate experiments. *p<0.03, ** p<0.002, *** p<0.0002, **** p<0.0001.

Then, the expression of PD-L1 and MYC was measured in human LUAD cell line H1299 and A549 treated with cAMP pathway drugs (forskolin, zardaverine and SQ2253) to explore the effect of cAMP pathway on these two genes. JQ1 and IFN-γ that known to regulate PD-L1 were used as controls. The gene and protein expression of PD-L1 were detected by qRT-PCR and flow cytometry. The results of qRT-PCR showed that forskolin (an adenylate cyclase activator) and zardaverine (a PDE4 inhibitor), which enhance cAMP pathway, decreased PD-L1 expression while SQ2253 (an adenylate cyclase inhibitor that inhibits the cAMP pathway) increased PD-L1 expression in H1299 and A549 cells (**Figure 4A**). The results of flow cytometry showed that the change of protein expression of PD-L1 was consistent with the gene expression in H1299 cells (**Figure 4B**). In a time-course experiment, the decrease of gene expression of PD-L1 and MYC in the H1299 cell line was quick, as seen at 2 h treatment with forskolin. The changes of PDL1 and MYC remained consistent at the various time points (**Figure 4D**). MYC regulation by these drugs was positively correlated with PD-L1 regulation (**Figures 4C**, **E**).

## Discussion

The cAMP/PDE4 axis has a pronounced influence on the immune system (17, 18). In benign inflammatory lesions, PDE4 inhibitors inhibit immune cell infiltration (19) and the inflammatory response of macrophages and dendritic cells by promoting cAMP-dependent PKA-CREB signaling (20). The cAMP/PDE4 axis can promote T-cell activation, helps to establish the memory of cytotoxic T-cells (21–23), and also regulates the transcription and secretion of interleukin (IL)-6, IL-8, and IL-10 (14, 24). As a part of the immune system, anti-tumor immunity is bound to be affected by the cAMP pathway. Our study illustrated that the cAMP/PDE4 pathway has a regulatory effect on immune infiltration. Immune infiltration as a determinant of the tumor micro-environment plays an important role in tumor progression, prognosis, and immunotherapeutic response (25). Therefore, it was predicted that the cAMP/PDE4 pathway could affect tumor prognosis. In this study, low expression of PDE4B was associated with a projected better prognosis of LUAD. However, PDE4B is associated with a poor prognosis in DLBCL (26). PDE4 inhibitors have cytotoxic effects on A549 lung cancer cells (27), and PDE4 is a key regulator of normal and cancer epithelial cell proliferation. PDE4 may thus be effective for the treatment of chronic inflammation and cancer cell proliferation (28). The role of PDE4B as a prognostic and therapeutic target, however, differs between specific tumors. CREB, another downstream molecule of cAMP/PDE4, was higher expressed and associated with a poor prognosis in this study. The Human Protein Atlas analysis similarly found the same prognostic trend for liver cancer, endometrial cancer, and malignant melanoma (29). CREB has been proposed as a proto-oncogene that supports tumor initiation, progression, and metastasis (30). Overexpression and hyper-activation of CREB are frequently observed in cancer, whereas the inhibition of CREB affects proliferation and apoptosis (31). PDE4 and CREB have been proposed as possible therapeutic targets in patients with cancer. On the other hand, the prognostic analysis of gene expression is quite different in different databases. In this study, the Kaplan-Meier plotter database was used to analyze. However, it was found that the level of gene expression was not related to prognosis using UALCAN database. Further analysis shows that the data in the Kaplan-Meier plotter database includes not only TCGA, but also GEO and EGA. UALCAN database only included TCGA database. The number of patients included in the KM plotter database is larger than that in the UALCAN database, which is why I chose the KM plotter database and the reason for the differences in results.

PD-L1 is an important molecule involved in immune evasion and immune micro-environment. High PD-L1 expression is associated with a better therapeutic effect of PD-1/PD-L1 inhibitors (4). PD-L1 expression is regulated by multiple factors, including inflammatory cytokines, such as IFN-γ (32) and IL-17 (33), microRNAs, including mir155 (34) and mir-17-5 (35), gene amplification and translocation (36, 37), post-transcriptional regulation pathways, such as CDK4 and CMTM6 (38, 39), and oncogenes, including MYC and STAT3 (40). Notably, it was verified that the MYC oncogene has been shown to regulate PD-L1 (41–45). In this study, our findings support the notion that cAMP/PDE4 is closely related to PD-L1 and MYC, and reveal the cAMP/PDE4/CREB1/MYC/PD-L1 regulatory network and cAMP/PDE4 axis regulation of PD-L1 is related to MYC. Another study showed that cAMP inhibits MYC activity through the mTOR pathway in a PDE4-dependent manner in colorectal cancer cells. PDE4 is responsible for the degradation of cAMP, and MYC acts as a transcriptional activator of PDE4, maintaining a low level of cAMP in cells and promoting the survival of colorectal cancer cells (46, 47). In B-cell lymphoma, cAMP indirectly inhibits MYC expression and forms a positive feedback loop with MYC (48). CREB is an important transcription factor (31) that actively regulates MYC and confers resistance to PI3K/mTOR inhibitors (49). CREBBP further binds directly to MYC, acetylates H3 at Lys9 and lys14 of MYC, and regulates the transcriptional function (50). The above studies showed that the interaction of cAMP/PDE4 axis and MYC exist, which is consistent with our study. Only a few studies have found the other indirect regulatory effect of cAMP/PDE4 axis on PD-L1, and there is bidirectional regulation in different tumors through TAZ/TEAD, JAK/STAT, or cytokines (13, 14), which suggests that cAMP/PDE4 axis may regulate the expression of PD-L1.

Our results have implications for understanding the mechanisms by which cancer regulates PD-L1 expression and elicits immune evasion in LUAD. Our findings are consistent with previous results suggesting a role for both the cAMP pathway and MYC in PD-L1 regulation. Future studies are needed to directly analyze the interactions among cAMP, PDE4, CREB1, CREBBP, MYC, and PD-L1.

## Conclusions

We demonstrated a potentially novel regulatory role of cAMP on PD-L1 through the cAMP/PDE4/CREB1/MYC/PD-L1 regulatory network in LUAD. We suggest that the cAMP pathway genes PDE4B and CREB may play a role in the mechanism whereby LUAD evades the immune system, participating in immune infiltration and influencing the prognosis of LUAD.

## Data availability statement

The original contributions presented in the study are included in the article/supplementary material. Further inquiries can be directed to the corresponding author.

## Author contributions

JW: conceptualization, investigation, formal analysis, and writing original draft. LT: formal analysis, investigation. DF: conceptualization, investigation, writing original draft. MS: formal analysis. WZ: data duration and formal analysis. DF: conceptualization, investigation, reviewing original draft. XL: reviewing original draft. All authors read and approved the final manuscript.

## Funding

This study was supported by Science Research Fund of Hunan Health and Family Planning Commission (grant no. B2016117), Young fond project of Natural Science Foundation of Hunan Province (Grant no. 2018JJ3748) in China, and C.J.Huang Medical Fellowship at Stanford University in USA. Dr Tong and Dr. Felsher’s work was supported by Stanford SPARK program and NIH grant R35CA253180.

## Acknowledgments

The authors thank all staff at the department of Oncology of the Second Xiangya Hospital of Central South University, Changsha, China, and all the members of Dr. Felsher’s laboratory.

## Conflict of Interest

The authors declare that the research was conducted in the absence of any commercial or financial relationships that could be construed as a potential conflict of interest.

## Publisher’s note

All claims expressed in this article are solely those of the authors and do not necessarily represent those of their affiliated organizations, or those of the publisher, the editors and the reviewers. Any product that may be evaluated in this article, or claim that may be made by its manufacturer, is not guaranteed or endorsed by the publisher.
