# Supplementary material for: Cyclic adenosine monophosphate/phosphodiesterase 4 pathway associated with immune infiltration and PD-L1 expression in lung adenocarcinoma cells
Source: Front Oncol. 2022 Aug 1;12:904969. doi: 10.3389/fonc.2022.904969 (PMC9376450; doi:10.3389/fonc.2022.904969)
Supplement: Supplementary file 3 [file Table_1.docx]

Supplement table 1: The primer sequences of Genes.

| Prime | Forward | Reverse |
| --- | --- | --- |
| PD-L1 | GGCATTTGCTGAACGCAT | CAATTAGTGCAGCCAGGT |
| MYC | CTGCGACGAGGAGGAGAACT | GGCAGCAGCTCGAATTTCTT |
| CD47 | GGCAATGACGAAGGAGGTTA | ATCCGGTGGTATGGATGAGA |
| UBC | AGCCCAGTGTTACCACCAAG | ACCCAAGAACAAGCACAAGG |
