# Supplementary material for: Cyclic adenosine monophosphate/phosphodiesterase 4 pathway associated with immune infiltration and PD-L1 expression in lung adenocarcinoma cells
Source: Front Oncol. 2022 Aug 1;12:904969. doi: 10.3389/fonc.2022.904969 (PMC9376450; doi:10.3389/fonc.2022.904969)
Supplement: Supplementary file 4 [file Table_2.docx]

Fig 1a-b: First: http://ualcan.path.uab.edu/analysis.html

Then: choose TCGA, Enter gene symbols: PDE4B, CREB1

Next: choose lung adenocarcinoma

Last: point “explore”

Fig 1c-d: First: http://kmplot.com/analysis/index.php?p=service&start=1

Then: choose “Lung cancer”

Next: Enter gene symbols: PDE4B(203708_at) or CREB1(204312_x_at)

Last: choose adenocarcinoma(n=865) in Histology, point “Draw Kaplan-Meier plot”

Fig 1e: First: cistrome.shinyapps.io/timer

Then: choose “Gene”

Next: Enter gene: PDE4B or CREB1 in Gene Symbol; choose LUAD in Cancer Types.

Last: Submit

When I made this figure, the website system I used was Timer (cistrome.shinyapps.io/timer). Now it is upgraded to TIMER 2.0 (http://timer.comp-genomics.org). So, the data is slightly different. This situation is explained on the website.

Fig 2a: Go to CBioPortal (<http://www.cbioportal.org/>), we found genes closely related to PD-L1 in TCGA-LUAD. There are 1908 genes (See attached table 1).

The Kyoto Encyclopedia of Genes and Genomes (KEGG) (http://www.kegg.jp/) was used to display genes related to the cAMP pathway (See attached table 2).

A Venn diagram (http://bioinformatics.psb.ugent.be/webtools/Venn/) was used to determine the intersection of the different gene groups. There are 21 genes:

| ADCY3 |
| --- |
| ADORA1 |
| ADORA2A |
| ATP1A3 |
| ATP1B1 |
| ATP1B3 |
| CAMK4 |
| EDNRA |
| GNA12 |
| GRIN3A |
| MAP2K1 |
| NFKB1 |
| PDE4B |
| PDE4D |
| PIK3CD |
| PPP1R12A |
| PTGER2 |
| RAC2 |
| SUCNR1 |
| TIAMI |
| VAV1 |

Fig 2b: Go to <http://www.kegg.jp/>, then, go to“KEGG PATHWAY”, enter “cAMP”, choose “map04024”, enter the above 21 genes. You can find where the gene is in the cAMP pathway.

Fig 2c: CBioPortal (<http://www.cbioportal.org/>) was used to analyze the associations between genes in TCGA-LUAD.

Fig 3a, d, e: CBioPortal (<http://www.cbioportal.org/>), which is the online database of The Cancer Genome Atlas (TCGA), was used to analyze the associations between genes in The Cancer Genome Atlas Lung Adenocarcinoma (TCGA-LUAD).

Fig 3c: The PPI network was constructed using the STRING database (http://string-db.org). Chose multiple proteins, enter “CREB1, PDE4B, PRKACA, MYC”

Fig 3b, f, g, h: The Genecards database (https://www.genecards.org) was used to find the transcription factors of related genes. Enter “CD274” or “MYC”. Point “Cistromic (ChIP-Seq) regulation report from SPP (The Signaling Pathways Project) for CD274 or MYC”. You can find the transcription factors of CD274 or MYC.

Fig 4a: See attached table 3.

Fig 4b: See attached table 4.

Fig 4c-e: See attached table 5-6.

Fig 4d: See attached table 7. The statistical analysis is done in PRISM 8 software, using the statistical method of one sample t test.
